# Trends in South Korean antimicrobial use and association with changes in *Escherichia coli* resistance rates: 12-year ecological study using a nationwide surveillance and antimicrobial prescription database

**DOI:** 10.1371/journal.pone.0209580

**Published:** 2018-12-31

**Authors:** Young Ah Kim, Yoon Soo Park, Taemi Youk, Hyukmin Lee, Kyungwon Lee

**Affiliations:** 1 Department of Laboratory Medicine, National Health Insurance Service Ilsan Hospital, Goyang, South Korea; 2 Department of Internal Medicine, National Health Insurance Service Ilsan Hospital, Goyang, South Korea; 3 Research Institute, National Health Insurance Service Ilsan Hospital, Goyang, South Korea; 4 Department of Laboratory Medicine and Research Institute of Bacterial Resistance, Yonsei University College of Medicine, Seoul, South Korea; Nitte University, INDIA

## Abstract

The purpose of this study is to determine the correlation between use of antimicrobials, such as fluoroquinolone, cefoxitin, and cefotaxime, and *Escherichia coli* resistance using a nationwide database. Nationwide data on antimicrobial consumption for 12 years (2002 to 2013) were acquired from a database of subjects (n = 1,025,340) included in the National Health Insurance Service-National Sample Cohort. National antimicrobial resistance rates of *E*. *coli* were obtained from the Korean Antimicrobial Resistance Monitoring System, which has been administered by the Korean Centers for Disease Control and Prevention since 2002. Fluoroquinolone-resistance rates of *E*. *coli* isolated from general hospitals have continuously increased since 2002 and were correlated with nationwide fluoroquinolone use (r = 0.82, *P* = 0.0012) or ciprofloxacin use (r = 0.90, *P*<0.0001). Cefotaxime-resistance rates of *E*. *coli* isolated from general hospitals markedly increased since 2008 and were correlated with nationwide cefotaxime use (r = 0.94, *P*<0.0001) or third-generation cephalosporin use (r = 0.96, *P*<0.0001). Cefoxitin-resistance rates of *E*. *coli* isolated from general hospitals peaked in 2010 and significantly correlated with cephamycin use at a two-year interval (r = 0.64, *P* = 0.0256). In conclusion, consumption of antimicrobials such as fluoroquinolone, cefoxitin, and cefotaxime is well correlated with the resistance rates of *E*. *coli* to these agents. This study provides background data for national antimicrobial management policies to reduce antimicrobial resistance.

## Introduction

Appropriate antimicrobial use is essential to control the development of microbial resistance. Higher rates of resistance have been reported in high antimicrobial consuming countries [1]. The volumes of antimicrobials used, along with the spread of resistant micro-organisms and genes encoding for resistance, are known to drive antimicrobial resistance. Although antimicrobial use and resistance rates are clearly related [2–4], correlation is not always obvious because of the complexity of resistance ecology [5–7]. Antimicrobial use and resistance rates are not perfectly correlated at national levels or across countries because resistance is affected by several factors, such as the proportion of individuals exposed, the proportion of children in the population, population density, the effects of different drug classes, and differences between bacterial species [8].

Because antimicrobial selective pressures differ among countries not only due to different resistance rates, but also because of discrepancies in medical systems, economic status, and sociological backgrounds, there is a need to investigate the correlation between antimicrobial use and resistance in each unique situation. Although associations between antimicrobial resistance and antimicrobial use according to geographic difference have been reported [1,4], studies on association between antimicrobial resistance and changes in antimicrobial use for long periods are scarce.

Antimicrobial resistance in South Korea is higher than in advanced regions such as the United States and Europe [9–11]. In South Korea, it is easy to obtain medical service since national health insurance for all populations started in 1989, and more antimicrobials are used than in the United States or Europe because prescription practices in clinics are not strictly regulated [12,13].

In our 12-year ecological study using a nationwide surveillance and antimicrobial prescription database, we investigated the correlation between changes in use of third-generation cephalosporin, cephamycin, and fluoroquinolone and *Escherichia coli* resistance rates. We focused on *E*. *coli* because the wide spread multidrug-resistant *E*. *coli* in community and healthcare facilities has become a serious situation in South Korea [14]. We hope this study will provide background data for the management of national antimicrobial use to reduce antimicrobial resistance.

## Materials and methods

### Ethics statement

The study was approved by National Health Insurance Service Ilsan Hospital IRB (NHIMC 2017-03-027). All methods were performed in accordance with the relevant guidelines and regulations. This study used National Health Insurance Service-National Sample Cohort with permission (REQ0000007624).

### Study population

Antimicrobial use in South Korea for 12 years (2002 to 2013) was acquired from a database of subjects from the National Health Insurance Service-National Sample Cohort (NHIS-NSC), a population-based cohort established to provide public health researchers and policy makers with representative, useful information regarding citizens’ utilization of health insurance and health examinations [15]. Informed consent for study participation was exempted according to local institutional review board policy. These data consist of a national sample of 1,025,340 people (male: 513,258, female: 512,082) which account for 2.2% of the total South Korean population as of the 2002 census.

### Nationwide antimicrobial consumption data

After separation of dispensing and prescribing function policy has been compelled by law in 2000 and all antimicrobials should be used by prescriptions which is electronically transferred to national health insurance service and accumulated in systemic database in South Korea [16]. Therefore, the data precisely reflect the entire use of antibiotics in South Korea. Systemic antimicrobial prescriptions based on the Anatomical Therapeutic Chemical Classification System (ATC) were included in this study, and amount of use was standardized by Daily Defined Dose (DDD) by expressing data in DDD per 1000 inhabitants daily (DID). This describes the number of people (per a population of 1,000) who use an antimicrobial every day and is an easy way to standardize antimicrobial use, considering dose, form, and frequency of use across antimicrobial products. Ten DID signifies that 1% of the population, on average, might receive a certain drug or group of drugs daily. Antimicrobial use by active ingredient (DDD/1,000 inhabitants/day, DID) can be calculated by following equation [17].

$$\frac{Amount\;of\;antimicrobials\;used\;for\;a\;year\;(mg)}{DDD\;(mg)\times 365\;days\times sample\;population\;of\;the\;year}\times 1,000\;inhabitants$$

### National antimicrobial resistance data

The data of antimicrobial resistance rates in South Korea were obtained from the Korean Antimicrobial Resistance Monitoring System [14], which has been performed by Korean Centers for Disease Control and Prevention since 2002. These data were obtained from non-duplicated clinical isolates in more than 100 bed-sized general hospitals distributed over all regions of South Korea. Antimicrobial susceptibility tests were done with disk diffusion method or automated minimal inhibition concentration methods. The results were interpreted according to Clinical and Laboratory Standard Institute guidelines [18]

### Statistical analysis

SAS software, version 9.2 was used for the statistical analysis (SAS Institute, Cary, NC, USA). The correlations between antimicrobial use and resistance were evaluated by correlation analysis, and a p value less than 0.05 was regarded as significant.

## Results

### Correlation between fluoroquinolone use and resistance rates to fluoroquinolone in *Escherichia coli*

**Fig 1:**
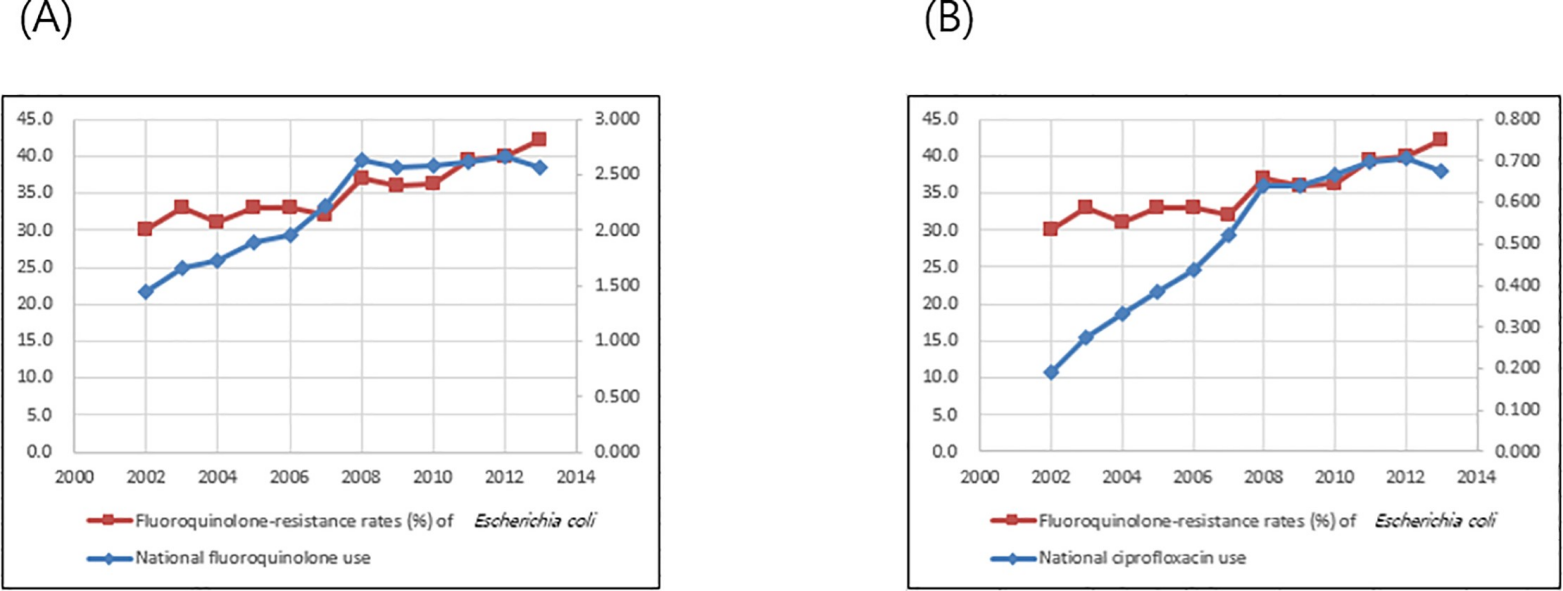
Correlation of nationwide fluoroquinolone use and resistance rates to fluoroquinolone of *Escherichia coli* (FQN-R-ECO), isolated from general hospitals from 2002 to 2013. (A) Correlation of total fluoroquinolone use and rates of FQN-R-ECO (Correlation coefficient r = 0.82, *P* = 0.0012); (B) Correlation of ciprofloxacin use and rates of FQN-R-ECO (Correlation coefficient r = 0.90, *P*<0.0001).

Total fluoroquinolone prescriptions in South Korea increased from 1.445 DID in 2002 to 2.565 DID in 2013. Ciprofloxacin prescriptions in South Korea increased from 0.191 DID in 2002 to 0.675 DID in 2013. Fluoroquinolone-resistance rates of *E*. *coli* isolated from general hospitals has continuously increased from 30% in 2002 to 42.1% in 2013 and nationwide fluoroquinolone use increased from 1.445 DID in 2002 to 2.565 DID in 2013. Fluoroquinolone-resistance rates of *E*. *coli* correlated with nationwide total fluoroquinolone use (r = 0.82, *P* = 0.0012) and ciprofloxacin use (r = 0.90, *P*<0.0001, **Fig 1**).

### Correlation between third- and fourth-generation cephalosporin and fluoroquinolone use and resistance rate to cefotaxime in *Escherichia coli*

**Fig 2:**
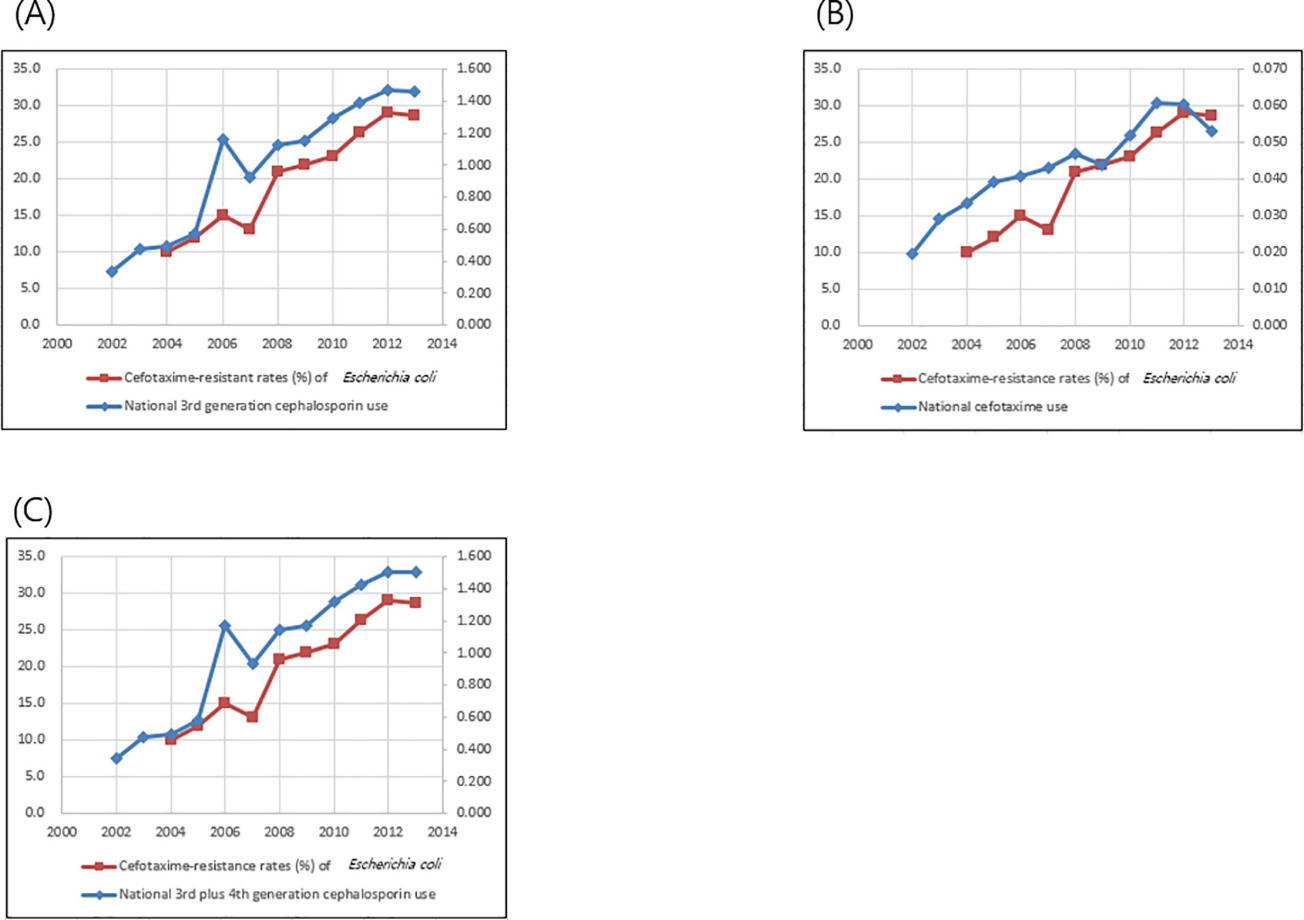
Correlation of nationwide cephalosporin/fluoroquinolone use and resistance rate to cefotaxime of *Escherichia coli* (CTX-R-ECO), isolated from general hospitals from 2002 to 2013. (A) Correlation of total third-generation cephalosporin use and rate of CTX-R-ECO (Correlation coefficient r = 0.96, *P*<0.0001); (B) Correlation of cefotaxime use and rate of CTX-R-ECO (Correlation coefficient r = 0.94, *P*<0.0001); (C) Correlation of total third- plus fourth-generation cephalosporin use and rate of CTX-R-ECO (Correlation coefficient r = 0.99, *P*<0.0001).

Total third-generation cephalosporin prescriptions in South Korea increased from 0.338 DID in 2002 to 1.457 DID in 2013. Cefotaxime prescriptions in South Korea increased from 0.020 DID in 2002 to 0.053 DID in 2013. Total fourth-generation cephalosporin prescriptions in South Korea increased from 0.002 DID in 2002 to 0.046 DID in 2013. Cefotaxime-resistance rate of *E*. *coli* isolated from general hospitals has continuously increased from 10% in 2004 to 28.7% in 2013. Cefotaxime-resistance rate correlated with nationwide third-generation cephalosporin use (r = 0.96, *P*<0.0001) and cefotaxime use (r = 0.94, *P*<0.0001). Cefotaxime-resistance rate of *E*. *coli* also correlated with summation of nationwide third- and fourth-generation cephalosporin use (r = 0.99, *P*<0.0001, **Fig 2**).

### Correlation between cephamycin use and resistance rates to cefoxitin in *Escherichia coli*

**Fig 3:**
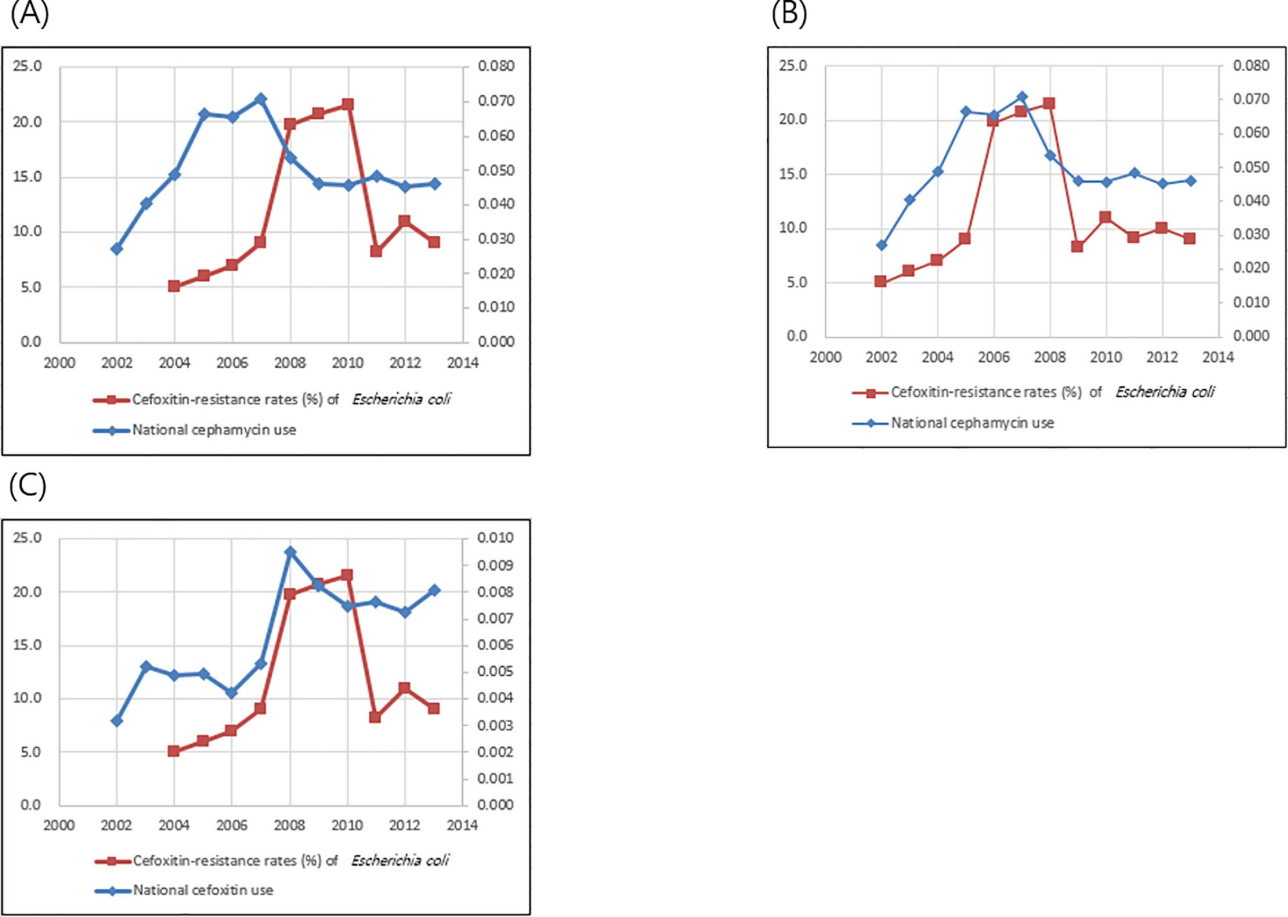
Correlation of nationwide cephamycin use and resistance rate to cefoxitin of *Escherichia coli* (FQX-R-ECO), isolated from general hospitals from 2002 to 2013. (A) Correlation of total cephamycin use and rate of FOX-R-ECO (Correlation coefficient r = -0.61, *P* = 0.0600); (B) Correlation of total cephamycin use and rate of FOX-R-ECO at two-year intervals (Correlation coefficient r = 0.64, *P* = 0.0256); (C) Correlation of cefoxitin use and rate of FOX-R-ECO (Correlation coefficient r = 0.73, *P* = 0.0158).

Total cephamycin use was 0.027 DID in 2002 and peaked at 0.071 DID in 2007. After 2007, cephamycin use decreased to 0.046 DID in 2013. Cefoxitin use was 0.003 DID in 2002 and increased to 0.010 DID in 2008. After 2008, cefoxitin use decreased to 0.008 DID in 2013. Cefoxitin-resistance rates of *E*. *coli* isolated from general hospitals peaked in 2010. This did not correlate with nationwide cephamycin use (r = 0.61, *P* = 0.0600), but significantly correlated with cephamycin use at a two-year interval (r = 0.64, *P* = 0.0256, **Fig 3A and 3B**). Cefoxitin-resistance rate of *E*. *coli* correlated with nationwide cefoxitin use (r = 0.73, *P* = 0.0158, **Fig 3C**).

### The correlation between cephamycin use and the resistance rate to cefoxitin in *Escherichia coli*

Total cephamycin use was 0.027 DID in 2002, and peaked to 0.071 DID in 2007. After 2007, cephamycin use decreased to 0.046 DID in 2013. Cefoxitin use was 0.003 DID in 2002, and increased to 0.010 DID in 2008. After 2008, cefoxitin use decreased to 0.008 DID in 2013. Cefoxitin-resistance rates of *E*. *coli* isolated from general hospitals have peaked in 2010 and decreased after that, which did not correlate with nationwide cephamycin use (r = 0.61, *P* = 0.0600), but significantly correlated with cephamycin use at 2 year interval (r = 0.64, *P* = 0.0256, **Fig 3A and 3B**). Cefoxitin-resistance rates of *E*. *coli* correlated with nationwide cefoxitin use (r = 0.73, *P* = 0.0158, **Fig 3C**).

## Discussion

We identified significant correlations between third-generation cephalosporin, cephamycin, and fluoroquinolone use and *E*. *coli* resistance in a 12-year ecological study using nationwide surveillance and an antimicrobial prescription database.

Previous antimicrobial exposure is a well-known risk factor for infection with ESBL- or AmpC-producing *E*. *coli* regardless of healthcare-associated or community-associated infections [19–21]. Single center or multicenter studies of correlation between antimicrobial consumption and resistance rates at the facility level could reflect selections of resistant organisms or acquisition of resistant genes in normal colonizers under antimicrobial pressure [22,23]. This data can be used as a background for antimicrobial stewardship programs to reduce unnecessary antimicrobial use and enhance appropriate treatment [24]. The spread of major antimicrobial-resistant pathogens to the community is an emerging problem [25–30], and all antimicrobial use, both in healthcare facilities and the community, contributes to enhancement of resistance [23].

Resistance to third-generation cephalosporins such as cefotaxime or ceftazidime via extended-spectrum β-lactamase (ESBL)-producing *E*. *coli* has been widespread in hospitals around the world since the late 1980s [31], but a sudden worldwide increase in the mid-2000s as mainly due to sequence type (ST) 131 with resistance to fluoroquinolone and third-generation cephalosporin with CTX-M–type ESBLs [32,33]. A recent study of molecular epidemiology showed that *H30Rx* subsets within the ST131-O25-H30 subclone were associated specifically with fluoroquinolone resistance, and CTX-M-15 was widely detected in South Korea [34], the spread of which is an important mechanism of *E*. *coli* resistance. *E*. *coli* is a major pathogen involved in urinary tract infection and shows good clinical outcomes [20,35]. However, severe life-threating infections, such as community-onset bacteremia by ESBL-producing *E*. *coli*, have increased in South Korea, becoming a concerning problem [34,36,37]^.^

It is well-known that a single ST131 strain rapidly expanded and disseminated in most current fluoroquinolone-resistant *E*. *coli* clinical isolates, which shares ESBL and mutations within the fluoroquinolone resistance-determining regions of *gyrA* and *parC* [38]. Therefore, co-selection of third-generation cephalosporin and fluoroquinolone may contribute to the increase in fluoroquinolone and cefotaxime resistance. Cephamycin is not a substrate of ESBL, and resistance to cefoxitin is mediated with AmpC β-lactamase, which results from overexpression of the chromosomal AmpC gene or acquisition of plasmid-mediated AmpC (pAmpC) determinant [39]. Since *Klebsiella pneumoniae* with transferrable pAmpC genes was first detected in South Korea in 1988 [40], pAmpC β-lactamase-producing *E*. *coli* with cefoxitin resistance has increased in South Korea [41].

Cefoxitin-resistance rates of *E*. *coli* isolated from general hospitals have markedly decreased since 2011 in South Korea, regardless of clinical breakpoint changes in CLSI guidelines 2010/2011 [42]. In this study, cefoxitin use decreased after 2008, but resistance rates of *E*. *coli* to cefoxitin did not decrease until 2011. Cefoxitin-resistance rates of *E*. *coli* did not significantly correlate with nationwide cephamycin use (r = -0.61, *P* = 0.0600), but showed significant correlation with nationwide cephamycin use (r = -0.64, *P* = 0.0256) at a two-year interval.

Although the time lag between antibiotic consumption and changes in resistance patterns on a national level is unknown, a period of one to two years has been suggested by other ecologic studies [1,4,43]. We suspect that response to cephamycin antibiotic pressure requires one to two years to influence cephamycin resistance rates, in accordance with previous studies [1,4,43].

The strong point of this study is analysis based on nationwide surveillance and an antimicrobial prescription database over a long period. Although associations between antimicrobial resistance and antimicrobial use according to geographic difference have been reported several times [1,4], studies about association between antimicrobial resistance and changes of antimicrobial usage for long periods are scarce. National Health Insurance Service (NHIS), a single-insurer system by the government of South Korea, maintains and stores all national records for healthcare utilization and prescriptions. NHIS developed the ‘National Health Information Database’ (NHID) for academic interests, containing personal information, demographics, and medical treatment data for South Korean citizens, which was generated using participants’ medical bill expenses claimed by medical service providers [15]. We used NHIS-NSC, a representative sample database by NHIS with a substantial volume of representative information, which provided large-scale, extensive, stable nationwide antimicrobial prescription data. This nationwide study does have a weak point in the significance of relatedness between antimicrobial consumption and resistance. But ecologically speaking, the density of antimicrobial usage involves the total amount of antimicrobial applied to a geographically defined number of individuals in any setting. Although this study has a less-defined study group compared to a case-control study, it can initiate a strategy of nationwide antimicrobial control in an evidence-based way.

In conclusion, correlations between use of antimicrobials such as fluoroquinolone, cefoxitin, and cefotaxime and *E*. *coli* resistance to these agents were observed in this study. This provides background data for national antimicrobial management policies to reduce antimicrobial resistance.

## Supporting information

S1 Table National antimicrobial consumption and resistance rate of *Escherichia coli*. (XLSX)
